# Cefepime-Induced Encephalopathy in a Patient With Uncompromised Renal Function: A Case Report

**DOI:** 10.1155/crdi/6650287

**Published:** 2025-03-29

**Authors:** Aayusha Dhakal, Ozone Gautam

**Affiliations:** ^1^Department of Internal Medicine, Kathmandu University School of Medical Sciences, Dhulikhel, Nepal; ^2^Department of Internal Medicine, Legacy Salmon Creek Medical Center, Mount Vista, Washington, USA

**Keywords:** case report, cefepime-induced neurotoxicity, metabolic encephalopathy, normal renal function, renal dose adjustment

## Abstract

Cefepime, a fourth-generation cephalosporin, is widely used in the treatment of infections caused by resistant Gram-negative bacteria especially *Pseudomonas aeruginosa*, or in severe infections, septic shock, and infections in immunocompromised patients. As it crosses the blood–brain barrier, it can cause neurotoxicity which has mostly been reported in patients with impaired renal function. Patients can present with drowsiness, confusion, delirium, agitation, stupor, or coma, and sometimes with generalized myoclonus and seizures within two to six days after starting the antibiotic. This is a rare case report where a patient with intact kidney function presented with confusion and incoherence. After excluding the possible causes for encephalopathy, cefepime-induced encephalopathy was diagnosed and the patient gradually improved after discontinuing the medication. This case is an unusual presentation of symptoms in a patient with normal kidney function, which necessitates further studies to establish other potential risk factors of cefepime-induced neurotoxicity.

## 1. Introduction

Cefepime, a cephalosporin antibiotic, is commonly used in the treatment of moderate to severe bacterial infections including pneumonia, urinary tract infections, and soft tissue infections [1]. While its neurotoxicity has been documented, it is very rare [2]. Although cefepime-induced neurotoxicity is usually reversible and resolves after withdrawing the treatment, prevention is the best approach, as patients may experience delirium and undergo unnecessary diagnostic tests and treatments. Here, we present a case of an elderly woman with a complex medical history who developed altered mental status following the administration of cefepime.

## 2. Case Presentation

A 72-year-old female with a history of COPD with chronic hypoxic and hypercapnic respiratory failure, recurrent aspiration pneumonia, lung adenocarcinoma in complete remission for six years, fibromyalgia on suboxone, hypothyroidism on levothyroxine, and iron deficiency anemia was admitted to our hospital. She had been experiencing repeated episodes of confusion and incoherence while treated with cefepime for pneumonia caused by *Klebsiella* and *Serratia*. She failed treatment with oral Bactrim (every 12 h for 7 days) following positive sputum cultures indicating these infections, and hence cefepime (every 8 h) was started after PICC line placement. After 7 doses, she developed intermittent confusion and incoherence, prompting her admission for altered mental status amid nonprogressive pulmonary symptoms.

The patient had a history of poorly differentiated adenocarcinoma with K-ras mutation of the right lung for which she completed chemoradiation therapy and was in complete remission for the last 6 years. The patient was on ipratropium, albuterol, and mometasone furoate for COPD. Chest X-ray on admission showed a patchy or ground-glass dense infiltrate involving the right upper lobe and lower half of the left lung which was stable compared to the chest X-ray done ten days ago. The patient also had a history of STEMI in the past that required a drug-eluting stent placement, and aspirin was continued. The last echocardiogram was done 3 years back which showed a left ventricular ejection fraction of 60%–65%. The patient was continued on olmesartan for hypertension once her blood pressure was within the baseline values. It was also reported that the patient had childhood epilepsy, which was treated with phenytoin, and was free of seizure activity for several years.

Upon arrival at our hospital, the patient was confused and exhibited repetitive incoherent speech. On examination, she was breathing comfortably with 2 L/min of oxygen (baseline) but was tachycardic and mildly hypotensive concerning sepsis, but no fever was reported. The patient was sleepy but responded to voice, oriented to self, and followed simple commands though she needed repetition at times. Pupils were round, regular, and reactive to light. Cranial nerve examinations were normal. There was no neck rigidity, and the plantar reflex was down going. On chest examination, there were coarse crackles bilaterally. Other systemic examinations were found to be normal.

**Figure 1:**
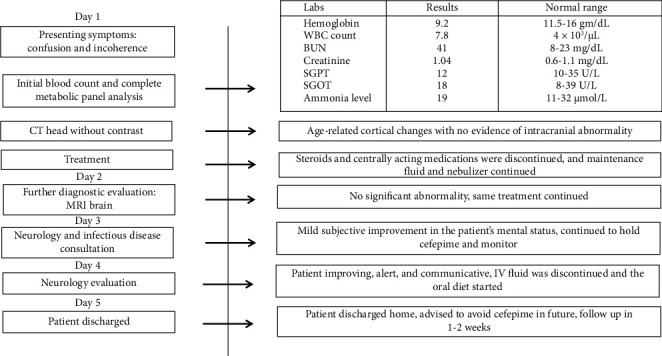
Timeline from the initial presentation of the patient's symptoms to their improvement and eventual discharge home.

Blood investigations showed a total leukocyte count of 7.8 × 1000/mL making infection unlikely (Figure 1). Blood venous gas showed mild CO2 elevation, but the complete metabolic panel and ammonia level were within normal limits excluding uremic and hepatic encephalopathy (Table 1). Additionally, the TSH level was within the normal range. A CT head showed age-related cortical atrophic changes without evidence of acute intracranial abnormality (Figure 1). MRI of the brain was done which revealed no acute infarct, acute intracranial hemorrhage, mass, hydrocephalus, midline shift, or abnormal post-contrast enhancement excluding any primary neurological causes (Figure 1). The case was also discussed with the experts in Infectious Diseases, and cefepime was discontinued due to concerns for cephalosporin-induced neurotoxicity (Figure 1). IV fluid was continued, and centrally acting medications like duloxetine and gabapentin were discontinued in hopes of improvement in mental status (Figure 1). Antihypertensives were held initially in the setting of low blood pressure. Suboxone for fibromyalgia, atorvastatin, and levothyroxine were continued as previously. On the second day of admission, there was a subjective improvement in the mental status of the patient, and on examination, the patient was oriented to self, place, and person. The case was then discussed with the Neurologist, and given symptomatic improvement following the discontinuation of cefepime, EEG was not recommended. Neurology advised that acute encephalopathy most likely was secondary to cefepime toxicity in the setting of slow clearance (Figure 1).

After careful assessment of history, examination findings, blood reports, and imaging findings, hepatic encephalopathy, uremic encephalopathy, infection, brain injuries, or any other neurological causes such as mass, lesion, or hydrocephalus were already excluded (Table 1). Hence, the diagnosis was pointed toward antibiotic-induced encephalopathy, cefepime in this case. On arrival to the probable diagnosis of cefepime-induced encephalopathy (CIE), antibiotics especially cefepime continued to be on hold. The patient was continued on IV fluids. Within 6 days of hospital admission, the patient continued to improve, with a return to her baseline mental status, stable pulmonary symptoms, and interval improvement in opacities in chest X-ray. The patient was discharged home with the advice not to resume cefepime and to follow up within 1-2 weeks.

## 3. Case Discussion

Metabolic encephalopathy encompasses a range of disorders arising from systemic imbalances in the body's normal metabolic processes. These disorders can arise from heterogeneous etiology leading to a clinical state of altered consciousness ranging from mild cognitive impairment to deep coma [3]. Out of many varying causes including toxins, organ failure, infectious diseases, electrolyte, and hormonal disbalances, drug-induced neurotoxicity is one of the reversible causes of encephalopathy [2]. In our case, cefepime, a fourth-generation cephalosporin used to treat bacterial pneumonia, was identified as the cause of encephalopathy through a diagnosis of exclusion. While encephalopathy induced by antibiotics is less frequent compared to that caused by immunosuppressive drugs, other antibiotics besides cephalosporins can also lead to this condition to varying extents, these include penicillins, carbapenems, aminoglycosides, quinolones, trimethoprim/sulfonamides, and metronidazole [4].

CIE was first reported in 1999 in a patient with end-stage renal disease (ESRD) on hemodialysis [5]. The mechanism of neurotoxicity due to cefepime was believed to be due to the drug's capacity to penetrate the blood–brain barrier, leading to a concentration-dependent competitive inhibition of *γ*-aminobutyric acid (GABA) [6]. Cefepime is largely excreted through the kidneys, with around 85% of the drug eliminated unchanged. In individuals with creatinine clearance of 10 mL/min, the half-life of cefepime is about five times longer than in those with normal kidney function [7]. As per the literature review, CIE was seen in patients with a CrCL under 60 mL/min when renal dose adjustments were not made [8]. CrCl in our patient was 68 mL/min before the administration of antibiotic, and hence decreased renal clearance may not be the only cause for CIE.

Few previous studies have considered the neurotoxic effects of cephalosporin antibiotics despite having normal renal function. In 2005, a case of a 79-year-old female with the diagnosis of pseudomonas urinary tract infection was treated with cefepime, following which the patient became acutely confused and the EEG revealed continuous generalized sharp and slow wave discharges. The patient was initially treated with anticonvulsant medications and once the cefepime was discontinued, the patient's mental status returned to baseline in 3 days [9]. Another case report from 2005 described a patient with normal renal function who experienced encephalopathy triggered by cefepime and cefixime. The acute delirium resolved during the hospital stay after the cephalosporins were discontinued. A study done in 2011 reported epileptiform discharges in 14 out of 1120 patients treated with cefepime, with the majority of cases occurring in individuals with normal renal function [9].

Similarly, a case report from 2016 presented a 76-year-old female with a past medical history of adrenal insufficiency, diabetes mellitus, hypertension, and hyperlipidemia admitted for hyponatremia and pulmonary infection with a normal renal function. She was found to have neurotoxicity secondary to cefepime use and her mental status returned to baseline after 3 days of discontinuing cefepime [9]. A case report in 2024 described a 79-year-old woman with a history of rectal cancer and depression who was hospitalized for a bacterial liver abscess. She developed encephalopathy following 11 days of treatment with cefepime and metronidazole. Her symptoms resolved quickly after discontinuing the antibiotics. Over the past 15–20 years, only a handful of cases of CIE have been reported in patients with normal renal function, making it an infrequent occurrence [10]. While CIE is a known side effect with an incidence of up to 15% in ICU patients, its presentation in individuals with normal renal function is even rarer [11].

Other known risk factors for cefepime-induced neurotoxicity are pre-existing brain injury, advanced age, high-dose therapy, and increased CNS penetration of cefepime [12]. Our patient had a history of epilepsy from childhood that was addressed solely in her early years which can be considered as one of the risk factors in this case.

According to the data from a recent meta-analysis, clinical symptoms of CIE include altered mentation, changes in consciousness, aphasia, and myoclonus. Altered mentation, which occurs in 47%–80% of cases, encompasses drowsiness, stupor, coma, and disorientation, manifested as confusion, delirium, and agitation, presenting a heterogeneous picture of the condition. Patients usually begin to exhibit neurotoxicity symptoms within 2–6 days of starting the medication [13, 14]. The mainstay of the treatment is discontinuing the culprit antibiotic and prompting clearance of the drug while providing supportive care and maintaining hemodynamic stability.

In our case, CIE was defined by the following criteria: (i) the presence of neurological symptoms and/or signs (such as confusion, altered consciousness, cognitive disturbances, and hallucinations) occurring after 2 days of cefepime therapy; (ii) no alternative explanation for the neurological symptoms and/or signs; and (iii) improvement (complete or partial resolution) of these symptoms within 2 days after stopping cefepime, without any other concurrent interventions [14]. Blood tests, urinalysis, head CT, and MRI brain were conducted to rule out other diagnoses. As the patient had taken 7 doses of IV cefepime, CIE was ruled in as the cause.

In such instances, a diagnosis is usually made by excluding other causes. The presence of triphasic waves with a high negative component (Tri-HNC) in EEG is a characteristic finding of CIE [15]. In this case, EEG was not recommended as the patient was already showing signs of improvement after holding cefepime. If necessary, a lumbar puncture can be performed, which may reveal elevated cephalosporin levels in the cerebrospinal fluid of affected patients [15]. As in this case, the antibiotic-induced encephalopathy is reversible and the patient will show improvement once the offending drug is stopped. While it is important to be mindful of this potential risk of cefepime, the concern must be weighed against the severity of the infection and the need for effective treatment.

## 4. Conclusion

Cephalosporin-induced encephalopathy is a rare condition. Once other causes of encephalopathy have been ruled out, CIE should always be considered as a possible diagnosis if a patient develops symptoms of neurotoxicity following cephalosporin administration as it can substantially change the prognosis. CIE is a diagnosis of exclusion with association of recent cefepime use. In cases like this, understanding the associated risk factors and judicious use of antibiotics are essential for preventing further complications.

## Figures and Tables

**Table 1:** Laboratory findings of the patient following admission.

Test	Reference range	Baseline	Day 1	Day 2	Day 3	Day 4
Sodium	136–145 mmol/L	140	140	140	140	140
Potassium	3.6–5.0 mmol/L	3.7	4.1	3.9	4.1	3.7
Chloride	98–107 mmol/L	97	98	100	99	100
Carbon dioxide	21–32 mmol/L	38	34	34	30	29
Anion gap	4–16 mmol/L	5	8	6	11	11
BUN	8–23 mg/dL	19	41	26	18	15
Creatinine	0.60–1.10 mg/dL	0.90	1.04	0.83	0.80	0.75
Glucose	82–99 mg/dL	103	110	91	92	86
Calcium	8.6–10.3 mg/dL	9.8	10.0	9.0	9.6	9.4

## Data Availability

Data supporting this case report are derived from patient medical records, which contain confidential information and cannot be shared due to privacy and ethical considerations.
